# Celiac Antigenicity of Ancient Wheat Species

**DOI:** 10.3390/foods8120675

**Published:** 2019-12-12

**Authors:** Maneka Malalgoda, Jae-Bom Ohm, Senay Simsek

**Affiliations:** 1 Department of Plant Sciences, North Dakota State University, Fargo, ND 58108-6050, USA; maneka.malalgoda@ndus.edu; 2 USDA-ARS, Edward T. Schafer Research Center, Cereal Crops Research Unit, Hard Spring and Durum Wheat Quality Laboratory, Fargo, ND 58108-6050, USA; jae.ohm@ars.usda.gov

**Keywords:** ancient grains, gliadin, celiac epitopes, proteomics

## Abstract

Ancient grains have gained renewed interest in the last few years due to their perceived nutritional benefits. The goal of this study was to examine the presence of celiac epitopes in different ancient grains and determine differences in the gliadin protein profile of such grains. To investigate celiac epitopes, an untargeted mass spectrometric method was used, and the gliadin protein profile was studied using reverse phase-HPLC. Our findings show that celiac epitopes can be detected in wheat-related ancient grains, such as einkorn, emmer, and Kamut, indicating that these ancient grains have the potential to elicit the immune response associated with celiac disease. Additionally, the results showed that the gliadin protein composition is significantly different between ancient grain species, which could result in varying functional properties in end-use applications.

## 1. Introduction

In recent years, there has been increased interest in the consumption of ancient grains, especially due to perceived health benefits compared to consuming modern wheat species. However, recent studies have shown that modern wheat cultivars are not significantly different in terms of composition or health benefits in comparison to ancient grains [1]. Nevertheless, decreased amounts of mineral micronutrients were noted by Shewry et al. [2,3].

Celiac disease develops in genetically susceptible individuals and is an autoimmune disease prevalent in 0.71% of the population in the United States [4]. Gluten forming proteins and its homologous found in cereals, such as wheat, rye, and barley, trigger the immune reaction which gives rise to celiac disease. The celiac antigenicity of ancient grains has long been debated, and thus far, studies show varied results. For instance, diploid wheat species with the AA genome, *Triticum monococcum,* has been found unsafe for consumption by celiac patients [5], although some studies have suggested otherwise [6]. Ancient tetraploid wheat species have also been analyzed in this manner, and studies have shown that ancient durum wheat species found in Italy are not celiac safe [7]. In recent studies by Dubois et al. [8,9] and Escarnot et al. [10], the celiac immunogenicity of spelt and hexaploid wheat was investigated where they found that there is large variation among different spelt accessions regarding their celiac reactivity. In these studies, various methods such as ELISA were used for the quantitative analysis of celiac epitopes.

Previous work on celiac antigenicity has shown that gliadin proteins, high molecular weight glutenin subunits, and low molecular weight glutenin subunits are associated with triggering the immune system in celiac subjects. Wieser and Koehler [11] reported that most of the antigenic sequences associated with celiac disease occur in the N-terminal domain of α-gliadin proteins, which mainly consist of glutamine, proline, and aromatic amino acids. Thus, the gliadin proteins can be considered as highly antigenic in comparison to other protein components in wheat.

The possibility of producing celiac safe wheat has been explored in many previous studies. The genetic variation amongst cultivars can be exploited to find accessions that are low in antigenicity, which can be used as parental varieties in breeding efforts as summarized by Malalgoda et al. [12]. Genome editing techniques, such as zinc-finger nucleases, transcription activator-like effector nucleases (TALEN), and clustered regularly interspaced short palindromic repeat (CRISPR)/Cas9 also have the potential to be used to develop celiac safe wheat. Downregulating genes that give rise to gliadin proteins can also be used to develop celiac safe wheat. Recently, ultra-low gluten barley was produced using traditional breeding techniques where three recessive alleles were combined to develop parental barley varieties with low hordein levels [13].

As mentioned above, there is conflicting information about the celiac safety of diploid and tetraploid wheat species, and to our knowledge, the gliadin protein profiles of ancient wheat species have not been analyzed in comparison to modern tetraploid and hexaploid wheat species. Thus, the objective of this study was to determine the presence of celiac epitopes using a proteomics approach and to characterize the gliadin protein composition in ancient diploid and tetraploid wheat species.

## 2. Materials and Methods

In this study, ancient grains, such as einkorn (*Triticum monococcum* L.), emmer (*Triticum dicoccum*), Kamut (*Triticum turgidum* subsp. *turanicum*), rye (*Secale cereale* L.), teff (*Eragrostis tef* (Zuccagni) Trotter) and sorghum (*Sorghum bicolor* Moench ssp. *Bicolor*), as well as pseudocereals such as amaranth (*Amaranthus L.*) and quinoa (*Chenopodium quinoa* Willd.) were analyzed for celiac epitopes and gliadin protein composition. The rye, teff and sorghum and the pseudocereals were kindly donated by Bay State Milling Company (Quincy, MA, USA). The einkorn, emmer, and Kamut samples were from Shiloh Farms (New Holland, PA, USA).

To determine the presence of celiac epitopes, an untargeted mass spectrometric approach was used as previously described by Malalgoda et al. [14]. Gliadin proteins were extracted using a modified Osborne fractionation method where 750 µL of 70% ethanol was added to 250 mg of flour. The samples were kept shaking at 1400 rpm for 1 hour at 30 °C in a heating block (Eppendorf Thermomixer R, Eppendorf AG, Hamburg, Germany). The samples were then centrifuged at 4550 g for 10 min (Eppendorf Centrifuge 5415C, Eppendorf AG, Hamburg, Germany) and filtered through a 0.45 µm nylon filter (VWR International, Radnor, PA, USA). After, the BCA assay (Peirce™ BCA assay kit, Thermo Scientific, Waltham, MA, USA) was performed to determine the protein content of the extracts. To prepare samples for SDS-PAGE, a volume of sample containing 40 µg of protein was mixed with SDS-PAGE sample buffer and boiled for 5 min. The samples were electrophoresed for 15 min at 55 V on an 8% Tris-Tricine gel. Afterwards, the gel was fixed using a solution containing 45% methanol (*v*/*v*), 45% water, (*v*/*v*) and 10% acetic acid (*v*/*v*). The gel was then stained overnight with Colloidal Coomassie Blue. The following day, gel bands were excised using a sterile blade and stored at −20 °C until mass spectrometric analysis.

The extracted proteins were digested (in-gel) with chymotrypsin, as previously described by Shevchenko et al. [15], and desalted using STAGE (Stop And Go Extraction) tip prior to mass spectrometric analysis. Approximately 1.5 µg of digested protein sample was analyzed on an Orbitrap Velos system (Thermo Fisher Scientific, Waltham, MA, USA) according to the method of Lin-Moshier et al. [16] with modifications as described by Malalgoda et al. [14]. The obtained data was analyzed against the nr_triticinae database (except amaranth and quinoa) and RefSeq_pentapentalae (for amaranth and quinoa) database downloaded from NCBI using the PEAKS 8.0 software (Bioinformatics Solutions, Waterloo, ON, Canada). In the analysis, the presence of celiac epitopes previously listed by Sollid et al. [17] was determined.

To analyze the gliadin protein composition of ancient wheat species and rye, the method of Malalgoda et al. [14] was used, where gliadin proteins were extracted according to the previously described method and analyzed using an Agilent Zorbax 300SB-C18 (4.6 × 250 mm, 5 µm) reverse phase column (Agilent technologies, Waldbroann, Germany). For the mobile phase, two solvents, A (95% water (*v*/*v*), 5% acetronitrile (*v*/*v*) and 0.1% trifluoroacetic acid (*v*/*v*)) and B (100% acetonitrile with 0.08% trifluoroacetic acid (*v*/*v*)) were used at a multi-step gradient as described by Malalgoda et al. [14]. The flow rate was 1 mL/min and the injection volume was 23 µL. The analysis was done at 65 °C and the absorbance was detected at 210 nm. The HPLC data was processed using the MATLAB (2018, The MathWorks, Natick, MA, USA) and statistical analysis was performed using SAS 9.4 (SAS Institute, Cary, NC, USA).

## 3. Results and Discussion

When analyzing the proteomics data for the presence of celiac epitopes, the celiac epitopes previously described by Sollid et al. [17] were used. Table 1 summarizes the celiac epitopes detected in the different samples that were used in this study. Celiac epitopes were detected in all three wheat-related ancient grains (einkorn, emmer, and Kamut) analyzed in addition to rye, which is known to elicit the immune response associated with celiac disease. As expected, celiac epitopes were not detected in teff and sorghum as well as the pseudocereals, which are considered gluten-free and safe for celiac subjects. A third of the epitopes evaluated were detected in einkorn and four epitopes were found in Kamut, which are diploid and tetraploid wheat species, respectively, consisting of the AA genome and the AABB genome. In emmer, which is a tetraploid species (AABB), only one epitope was detected. However, this could be due to limitations in the method or the heterogeneity of the sample and does not imply that only this epitope is found in emmer. It is interesting to note that the FRPQQPYPQ (glia-α20) epitope was detected in all three ancient wheat species while the glia-α9 epitope (PFPQPQLPY) and epitope QGSFQPSQQ were detected in two of the three species. In our previous work, the glia-α-20 epitope was the most prevalent epitope analyzed, whereas the glia-α9 epitope was the second most prevalent in historical and modern wheat cultivars released in North Dakota between 1910 and 2013 [9]. Three of the nine γ-gliadin epitopes were also found in the wheat species, although they were detected in a more random manner. The epitopes in ω-gliadin proteins were not detected in any of the ancient wheat species, which could be explained by the distribution of ω-gliadin T-cell epitopes encoded by the A/D genomes in comparison to the B genome [18]. In hexaploid bread wheat, celiac epitopes are distributed throughout the chromosomes encoding ω-gliadin in the A/D genomes, whereas the B-encoded ω-gliadins do not show T-cell epitopes. Thus, the lack of the D genome in diploid and tetraploid ancient wheat species could be associated with the non-detection of ω-gliadin celiac epitopes. Additionally, non-detection could be due to genetic variation between the different ancient grain cultivars. According to these results, celiac epitopes can be found in ancient wheat species, which make them unsuitable for consumption by celiac subjects. According to Sollid et al. [17], the proteins rich in glutamine and proline, collectively termed prolamins of rye are referred to as secalin proteins, and T-cell epitopes in secalin proteins are homologous to that of wheat [19]. In this analysis, the proteomics data were used to determine the presence of two T-cell epitopes in rye secalin proteins, where one of the two peptides was detected. All three wheat-related ancient grain species and rye fall under the *Poaceae* family which may explain why they contain celiac epitopes. In a few of the ancient grains, namely teff and sorghum, and in the pseudocereals that were analyzed, as expected, none of the celiac epitopes were detected, further confirming that they are celiac safe.

All ancient grains and pseudocereals used in the study belong to the division of Magnoliopsida. The ancient wheat species and rye are then categorized as monocots, whereas the pseudocereals are categorized as dicots and this is the point of taxonomic separation for these species.

**Figure 1:**
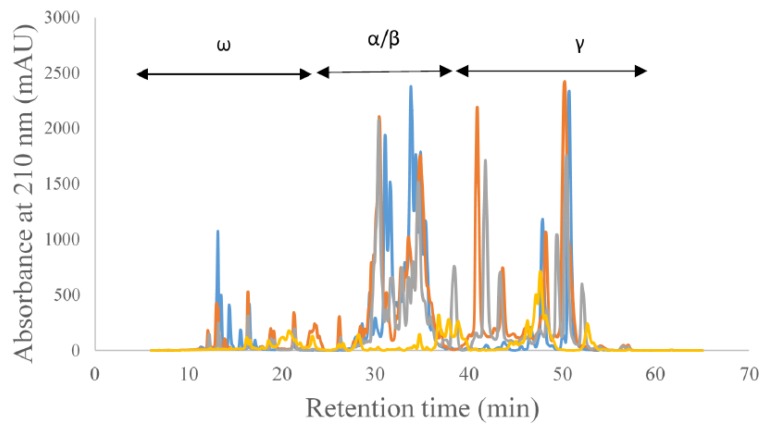
Reverse Phase-HPLC profiles of ancient grain species and the corresponding regions for different gliadin protein types.

Gliadin proteins can be separated into α/β, γ, and ω according to the electrophoretic mobility of the different protein fractions. As mentioned above, the α-gliadin fraction is considered to be highly antigenic compared to the other gliadin protein fractions. The different gliadin protein fractions have various roles in functionality as determined by previous studies. The separation of gliadin proteins into its different constituents can be achieved through reverse phase-HPLC (RP-HPLC). The gliadin/ prolamin protein profile of ancient wheat species and rye is shown in Figure 1, where the different gliadin types are separated according to previous work [20].

Using the data from the HPLC analysis, the percent area corresponding to individual gliadin proteins was determined for each sample, and significant differences in the percent area were analyzed. For all three gliadin protein types, the percent area values were significantly (*p* ≤ 0.001) different between the different ancient grains. For ω-gliadin proteins, rye showed the highest area percent (21%), whereas the Kamut showed the lowest (4%). On average, the percent area for the wheat-related ancient grains was less than half of that of rye. For α/β-gliadins, the highest area percent was found in einkorn (68%), whereas the lowest was found in rye (24%). In general, the area percent of the wheat-related ancient grains was more than double compared to that of rye. Therefore, when considering the abundance of α-gliadin proteins, rye may have lower celiac antigenicity. However, to produce celiac-safe rye, the antigenic epitopes need to be removed. The γ-gliadin protein percentage of the different species showed a trend similar to that of ω-gliadin proteins, with the highest percentage found in rye (55%), and the lowest in einkorn (23%).

## 4. Conclusions

Overall, this study shows that celiac epitopes can be found in wheat-related ancient grains, such as einkorn, emmer, and Kamut. The study also shows that such epitopes are present in rye, which is known to trigger the immune response associated with celiac disease. As expected, such epitopes were not found in the protein extractions from teff and sorghum, which are gluten free, as well as the pseudocereals. The analysis of the gliadin protein profile of the different ancient grains shows that the abundance of the different gliadin types varies widely between species, which could be indicative of differences in celiac antigenicity and functionality in food systems.

## Figures and Tables

**Table 1:** Analysis of celiac epitopes in ancient grains using untargeted mass spectrometry.

Immunogenic Peptide		Amaranth	Einkorn	Emmer	Kamut	Quinoa	Rye	Sorghum	Teff
Type of Gliadin	Sequence *								
Alpha gliadin	FRPQQPYPQ		✓	✓	✓				
	PFPQPQLPY		✓		✓				
	PYPQPQLPY								
	PQPQLPYPQ								
	QGSFQPSQQ		✓		✓				
Gamma gliadin	PQQSFPQQQ								
	IQPQQPAQL				✓				
	QQPQQPYPQ								
	SQPQQQFPQ								
	PQPQQQFPQ		✓						
	QQPQQPFPQ								
	PQPQQPFCQ								
	QQPFPQQPQ		✓						
Omega gliadin	PFPQPQQPF								
	PQPQQPFPW								
Omega secalin	PFPQPQQPF						✓		
	PQPQQPFPQ								

* Epitopes listed in Sollid et al. [17], ✓: Epitope detected.
